# The study of plain CT combined with contrast-enhanced CT-based models in predicting malignancy of solitary solid pulmonary nodules

**DOI:** 10.1038/s41598-024-72592-9

**Published:** 2024-09-19

**Authors:** Wenjia Zhang, Xiaonan Cui, Jing Wang, Sha Cui, Jianghua Yang, Junjie Meng, Weijie Zhu, Zhiqi Li, Jinliang Niu

**Affiliations:** 1 Department of Medical Imaging, Shanxi Medical University, NO.56 Xinjian Road, Taiyuan, 030000 Shanxi The People’s Republic of China; 2 National Clinical Research Center for Cancer, Key Laboratory of Cancer Prevention and Therapy, Tianjin, Department of Radiology, Tianjin Medical University Cancer Institute & Hospital, Tianjin, The People’s Republic of China; 3 Department of Epidemiology and Health Statistics, School of Public Health, Hangzhou Medical College, Hangzhou, The People’s Republic of China; 4 Department of Radiology, The Second Hospital of Shanxi Medical University, Taiyuan, The People’s Republic of China; 5 Department of Cardiothoracic Surgery, The Second Hospital of Shanxi Medical University, Taiyuan, The People’s Republic of China

**Keywords:** Pulmonary nodules, Logistic regression model, Prediction, Contrast-enhanced CT, Cancer models, Lung cancer, Cancer imaging

## Abstract

To compare the diagnostic performance between plain CT-based model and plain plus contrast CT-based modelin the classification of malignancy for solitary solid pulmonary nodules. Between January 2012 and July 2021, 527 patients with pathologically confirmed solitary solid pulmonary nodules were collected at dual centers with similar CT examinations and scanning parameters. Before surgery, all patients underwent both plain and contrast-enhanced chest CT scans. Two clinical characteristics, fifteen plain CT characteristics, and four enhanced characteristics were used to develop two logistic regression models: model 1 (plain CT only) and model 2 (plain + contrast CT). The diagnostic performance of the two models was assessed separately in the development and external validation cohorts using the AUC. 392 patients from Center A were included in the training cohort (median size, 20.0 [IQR, 15.0–24.0] mm; mean age, 55.8 [SD, 9.9] years; male, 53.3%). 135 patients from Center B were included in the external validation cohort (median size, 20.0 [IQR, 16.0–24.0] mm; mean age, 56.4 [SD, 9.6] years; male, 51.9%). Preoperative patients with 201 malignant (adenocarcinoma, 148 [73.6%]; squamous cell carcinoma, 35 [17.4%]; large cell carcinoma,18 [9.0%]) and 326 benign (pulmonary hamartoma, 118 [36.2%]; sclerosing pneumocytoma, 35 [10.7%]; tuberculosis, 104 [31.9%]; inflammatory pseudonodule, 69 [21.2%]) solitary solid pulmonary nodules were gathered from two independent centers. The mean sensitivity, specificity, accuracy, PPV, NPV, and AUC (95%CI) of model 1 (Plain CT only) were 0.79, 0.78, 0.79, 0.67, 0.87, and 0.88 (95%CI, 0.82–0.93), the model 2 (Plain + Contrast CT) were 0.88, 0.91, 0.90, 0.84, 0.93, 0.93 (95%CI, 0.88–0.98) in external validation cohort, respectively. A logistic regression model based on plain and contrast-enhanced CT characteristics showed exceptional performance in the evaluation of malignancy for solitary solid lung nodules. Utilizing this contrast-enhanced CT model would provide recommendations concerning follow-up or surgical intervention for preoperative patients presenting with solid lung nodules.

## Introduction

Lung cancer is the leading cause of cancer deaths in the world^1,2^. The imaging evaluation of a solitary pulmonary nodule (SPN) is complex, however, which can be improved by early detection and prompt treatment^3–8^. The American National Lung Screening Trial (NLST) 2011 showed that CT screening for lung cancer reduced mortality by 20% compared to chest X-rays^9^. Although plain CT is prominent in lung nodule detection, it is limited in differentiating benign from malignant^10–12^. To facilitate timely and personalized patient treatment, it is crucial to accurately characterize the nature of lung lesions. Performing a tissue biopsy is an invasive procedure, done especially on smaller nodules or in difficult-to-reach areas of the lung^13,14^. The PET-CT scan plays a crucial role in the diagnosis of pulmonary nodules. Nevertheless, it is associated with a notable incidence of false negatives, exemplified by cases where lung adenocarcinoma presents as subsolid nodules, as well as false positives, with pathological findings indicating inflammatory pseudotumors and tuberculosis. In addition, PET-CT requires expensive equipment and increases patients' financial burdens. In contrast to PET-CT, contrast-enhanced CT is relatively low-cost and remains the primary preoperative examination for most patients with lung nodules in developing countries. Contrast-enhanced CT helps to highlight blood vessels and other structures, making it easier to identify abnormalities such as tumors^15^. Lung nodule CT contrast enhancement reflects the nodule blood supply. The region without enhancement is strongly predictive of benign hypovascular lesion, and the region with a rich blood supply may reflect underlying nodule angiogenesis and indicate nodule malignancy^16–18^. Thus, a model for diagnosing lung nodules using contrast-enhanced CT is needed.

A study focusing on contrast-enhanced CT showed high sensitivity to differentiate benign and malignant nodules by using 15HU enhancement as a cut-off value (sensitivity 98%), however, the specificity for malignancy was only 50–60%^17^. These results showed that the only feature of enhancement value is not enough to effectively differentiate benign from malignant. In practical clinical work, radiologists will comprehensively consider the imaging features of nodule size, margin, and location, especially the heterogeneity of enhancement. This suggests that we need to incorporate more features to build the model, rather than just a single enhancement value feature.

This study aims to establish and authenticate two models that rely on plain CT and contrast-enhanced CT to predict the malignancy of solitary solid pulmonary nodules in a dual-center investigation. Through a comparative analysis of the diagnostic effectiveness of the two models, the study endeavors to elucidate the optimal preoperative diagnostic approach for solitary solid pulmonary nodules.

## Materials and methods

### Ethics approval

The study was conducted in compliance with the principles outlined in the Declaration of Helsinki and received approval from the Ethics Committees of the Tianjin Medical University Cancer Institute and Hospital (TMUCIH), and Second Hospital of Shanxi Medical University (SHSMU) (No. bc2021327), and all procedures adhered to pertinent guidelines and regulations. Informed consent was obtained from all participants.

A retrospective analysis of data from dual centers was conducted in this study. Between January 2012 and July 2021, 392 patients with pathologically confirmed solitary pulmonary nodules were recruited from center A, and 135 patients from center B. Both centers had the same inclusion and exclusion criteria: requiring patients to have a primary solitary solid lung nodule with a diameter less than 30mm on CT, to have received both plain CT and contrast-enhanced CT within one month before surgery, to have clear histologic types as indicated by postoperative pathology reports, and to have no metastasis. The study employed exclusion criteria consisting of four conditions: (1) solitary solid lung nodules with a diameter of 30mm or greater, (2) patients who underwent preoperative therapy such as neoadjuvant chemotherapy or radiotherapy, (3) patients with an unclear pathology result, and (4) unavailable contrast-enhanced CT images.

### Clinical and radiological data

We retrospectively collected and analyzed radiological data from two hospitals. The study enrolled a total of 392 patients at Center A and 135 patients at Center B who met the inclusion and exclusion criteria. Preoperative contrast-enhanced and plain CT scans were acquired using SOMATOM Sensation 64, GE Discovery 750HD, GE Revolution, and Philips IQon. RadiAnt DICOM Viewer (version 2021.1) was used for image evaluation, in a lung window setting (width, 1450HU; level, -500HU) and a mediastinal window setting (width, 350HU; level, -500HU) respectively. After mutual consultation, two radiologists (C.X.N 10-year experience and Z.W.J 9-year experience) resolved discrepancies between CT characteristics given by each radiologist. Two clinical characteristics (1) age; (2) gender, fifteen plain CT characteristics (3) diameter (4) nodule location_1 (left upper, left lower, right upper, right middle, right lower); (5) nodule location_2 (peripheral, central); (6)shape (round/oval, irregular); (7)margin (smooth, lobulated, spiculated); (8) calcification; (9) fat; (10) necrosis; (11) cavitation; (12) air bronchogram; (13) pleural indentation; (14) vascular invasion; (15) post obstructive pneumonia; (16) satellite nodules; (17) Plain CT value and four enhanced CT characteristics (18) subjective enhancement (uniform, heterogeneous, no); (19) enhanced CT value; (20) enhancement difference; (21) enhancement rate (enhancement difference / plain CT value).

### Computed tomography examination

CT examinations were performed at dual centers, utilizing the GE Discovery CT 750 HD and Siemens Somatom Sensation 64 CT system at the Center-A, and the GE Revolution CT, GE Discovery CT 750 HD, and Philips iQon spectral CT at the Center-B. The examinations consisted of an acquisition both with and without iodine contrast. The inspiratory scans are performed with the patient in the supine position from the apex to the base of the lungs. The scanning protocol was as follows: at Center-A, tube voltage 120 kVp with automatic tube current modulation. The iodine contrast agent Visipaque (Iodixanol, 270 mg/ml) was administered at an amount of 1.5 mL/kg and injection rate of 2.5 mL/s. Contrast agents were administered intravenously through the upper extremity. Scanning was performed 70 s after the start of the injection. At Center-B, tube voltage 120 kVp with automatic tube current modulation. The iodine contrast agent Visipaque (Iodixanol, 320 mg/ml) was administered at an amount of 60 ml and injection rate of 3.5 mL/s. Contrast agents were administered intravenously through the upper extremity. Scanning was performed 60 s after the start of the injection. The pitch, acquired slice thickness, and reconstructed slice thickness varied among the GE, Siemens, and Philips CT systems. Specifically, the GE CT system had a pitch of 0.984 and acquired and reconstructed slice thickness of 1.25 mm, the Siemens CT system had a pitch of 0.95 and acquired and reconstructed slice thickness of 1.5 mm, and the Philips CT system had a pitch of 1.23 and acquired and reconstructed slice thickness of 1.0 mm.

### Model construction and evaluation

Our two models for lung nodule malignancy classification were built using 392 nodules from Center-A as a training cohort and 135 nodules from Center-B as an external validation cohort. Clinical and CT characteristics were normalized using a feature standardization method^19^. Based on the training cohort, model 1 (plain CT only) was built with two clinical and fifteen plain CT characteristics based on logistic regression (*P* < 0.05 in univariable analysis). Model 2 (plain + contrasted) was built with two clinical, fifteen plain CT characteristics, and four enhanced CT characteristics. In the external validation cohort, we verify the classification efficiency of the two models.

### Statistical analysis

The present study utilized the Students' t-test to compare continuous data, which were expressed as mean values accompanied by standard deviation (SD), and categorical data, which were presented as percentages (%). Additionally, the Kolmogorov–Smirnov test was employed to compare non-normally distributed continuous variables, which were presented as medians with interquartile ranges (IQR). Statistical significance was determined by a p-value of 0.05 on both sides. The evaluation of the two cohorts involved the assessment of various performance metrics, including the area under the receiver operating characteristic curve (AUC), accuracy, sensitivity, specificity, negative predictive values (NPV), and positive predictive values (PPV). SPSS software version 20.0 (IBM Corp.) was used for the statistical analyses.

## Results

### Participants

A total of 527 preoperative patients with a solitary pulmonary solid nodule were gathered from two independent institutions in China, with a median size of 20.0 (IQR, 15.0–24.0) mm and a mean age of 55.9 (SD, 9.8) years, of which 52.9% were male. The training cohort comprised 392 patients from Center-A, with a median size of 20.0 (IQR, 15.0–24.0) mm, a mean age of 55.8 (SD, 9.9) years, and a male representation of 53.3%. The external validation cohort consisted of 135 patients from Center-B, with a median size of 20.0 (IQR, 16.0–24.0) mm, a mean age of 56.4 (SD, 9.6) years, and 51.9% male. All clinical and CT characteristics are summarized in Table 1.

**Table 1:** Characteristic baseline of patients in cohorts.

Characteristics	Total cohort (n = 527)	Training cohort (N = 392)	External validation cohort (N = 135)	*P*
Age, mean (SD), years	55.9 ± 9.8	55.8 ± 9.9	56.4 ± 9.6	0.879
Gender, n(%)				0.769
Female	248(47.1%)	183(46.7%)	65(48.1%)	
Male	279(52.9%)	209(53.3%)	70(51.9%)	
Histologic Types, n(%)				0.473
Malignant	201(38.1%)	153(39.0%)	48(35.6%)	
Adenocarcinoma	148(73.6%)	115(75.2%)	33(68.8%)	
Squamous Cell Carcinoma	35(17.4%)	23(15.0%)	12(25.0%)	
Large Cell Carcinoma	18(9.0%)	15(9.8%)	3(6.2%)	
Benign	326(61.9)	239(61.0%)	87(64.4%)	
Pulmonary Hamartoma	118(36.2%)	87(36.4%)	31(35.6%)	
Sclerosing Pneumocytoma	35(10.7%)	27(11.3%)	8(9.2%)	
Tuberculosis	104(31.9%)	74(31.0%)	30(34.5%)	
Inflammatory Pseudotumor	69(21.2%)	51(21.3%)	18(20.7%)	
Size, median (IQR), mm	20.0(15.0–24.0)	20.0(15.0–24.0)	20.0(16.0–24.0)	0.204
Location_1, n(%)				
Left Upper Lobe	122(23.1%)	96(24.5%)	26(19.3%)	
Left Lower Lobe	93(17.6%)	72(18.4%)	21(15.6%)	
Right Upper Lobe	165(31.3%)	122(31.1%)	43(31.9%)	
Right Middle Lobe	43(8.2%)	28(7.1%)	15(11.1%)	
Right Lower Lobe	104(19.7%)	74(18.9%)	30(22.2%)	
Location_2, n(%)				0.426
Peripheral	479(90.9%)	354(90.3%)	125(92.6%)	
Central	48(9.1%)	38(9.7%)	10(7.4%)	
Shape, n(%)				
Round/oval	337(63.9%)	251(64.0%)	86(63.7%)	
Irregular	190(36.1%)	141(36.0%)	49(36.3%)	
Margin, n(%)				0.981
Smooth	123(23.3%)	91(23.2%)	32(23.7%)	
Lobulated	213(40.4%)	158(40.3%)	55(40.7%)	
Spiculated	191(36.2%)	143(36.5%)	48(35.6%)	
Calcification, n(%)				0.624
No	463(87.9%)	346(88.3%)	117(86.7%)	
Yes	64(12.1%)	46(11.7%)	18(13.3%)	
Fat, n(%)				0.174
No	490(93.0%)	361(92.1%)	129(95.6%)	
Yes	37(7.0%)	31(7.9%)	6(4.4%)	
Necrosis, n(%)				0.482
No	491(93.2%)	367(93.6%)	124(91.9%)	
Yes	36(6.8%)	25(6.4%)	11(8.1%)	
Cavitation, n(%)				0.657
No	455(86.3%)	337(86.0%)	118(87.4%)	
Yes	72(13.7%)	55(14.0%)	17(12.6%)	
Air Bronchograms, n(%)				0.309
No	462(87.7%)	347(88.5%)	115(85.2%)	
Yes	65(12.3%)	45(11.5%)	20(14.8%)	
Pleural Indentation, n(%)				0.137
No	208(39.5%)	162(41.3%)	46(34.1%)	
Yes	319(60.5%)	230(58.7%)	89(65.9%)	
Vascular Invasion, n(%)				0.169
No	445(84.4%)	336(85.7%)	109(80.7%)	
Yes	82(15.6%)	56(14.3%)	26(19.3%)	
Postobstructive Pneumonia, n(%)				0.306
No	444(84.3%)	334(85.2%)	110(81.5%)	
Yes	83(15.7%)	58(14.8%)	25(18.5%)	
Satellite Nodules, n(%)				0.062
No	476(90.3%)	360(91.8%)	116(85.9%)	
Yes	51(9.7%)	32(8.2%)	19(14.1%)	
Subjective Enhancement, n(%)				0.054
No	152(28.8%)	109(27.8%)	43(31.9%)	
Uniform	34(6.5%)	20(5.1%)	14(10.4%)	
Heterogeneous	341(64.7%)	263(67.1%)	78(57.8%)	
CT value, mean (SD), HU				
Plain CT value	22.0 ± 16.2	21.5 ± 16.9	23.5 ± 13.7	0.210
Enhanced CT value	53.3 ± 30.5	53.0 ± 30.9	53.9 ± 29.7	0.920
Enhancement difference	31.2 ± 25.2	31.5 ± 24.9	30.4 ± 26.3	0.342
Enhancement Rate	2.1 ± 6.8	2.3 ± 7.5	1.5 ± 5.8	0.131

### The development of two models for predicting pulmonary nodule malignancy

Three hundred and ninety-two nodules (adenocarcinoma, 115 [75.2%]; squamous cell carcinoma, 23 [15.0%]; large cell carcinoma, 15 [9.8%]); pulmonary hamartoma, 87 [36.4%]; sclerosing pneumocytoma, 27 [11.3%]; tuberculosis, 74 [31.0%]; inflammatory pseudonodule, 51 [21.3%]) were in the training cohort. To compare the diagnosis difference between plain CT and enhanced CT for solid pulmonary nodules. We constructed two logistic regression models that without and with contrast enhanced CT characteristics. In the plain CT model, a total of twenty-one variables were generated, with vascular invasion (1.0877) and pleural indentation (0.5985) being the two most significant variables for predicting nodule malignancy, and fat (− 0.9334) and smooth (− 0.7732) being the two most important variables for predicting nodule benign. In the contrast-enhanced CT model, twenty-five variables were generated, with heterogeneous enhancement (1.8129) and vascular invasion (0.9249) being the two most significant variables for predicting nodule malignancy, and fat (− 0.9425) and round/oval (− 0.7041) being the two most important variables for predicting nodule benign. The detailed variables information of the two models are shown in Tables 2 and 3.

**Table 2:** The relative weight of model 1 (plain CT only) CT characteristics for predicting malignancy.

Feature	Coef	Mean	SD	Relative_to_max
Vascular_invasion	1.0877	0.1429	0.3499	1
Pleural_indentation	0.5985	0.5867	0.4924	0.5503
Age	0.5959	55.7806	9.8904	0.5479
Diameter(mm)	0.4301	19.4316	5.6907	0.3954
Air_Bronchograms	0.4159	0.1148	0.3188	0.3824
Location_2 (0 = Peripheral;1 = Central)	0.3606	0.0969	0.2959	0.3315
Gender (0 = woman; 1 = man)	0.2665	0.5332	0.4989	0.245
Postobstructive	0.2201	0.1480	0.3551	0.2024
Lobulated	0.1858	0.4031	0.4905	0.1709
Cavitation	0.1522	0.1403	0.3473	0.14
Plain_CT_Value	0.1308	21.5051	16.9014	0.1202
Left_upper	0.0762	0.2449	0.4300	0.0701
Left_lower	0.0453	0.1837	0.3872	0.0417
Right_middle	− 0.0036	0.0714	0.2575	− 0.0033
Right_upper	− 0.0918	0.3112	0.4630	− 0.0844
Necrosis	− 0.301	0.0638	0.2444	− 0.2767
Calcification	− 0.4927	0.1173	0.3218	− 0.453
Satellite_Nodules	− 0.6551	0.0816	0.2738	− 0.6023
Shape (0 = round/oval; 1 = irregular)	− 0.6764	0.3597	0.4799	− 0.6219
Smooth	− 0.7732	0.2321	0.4222	− 0.7108
Fat	− 0.9334	0.0791	0.2699	− 0.8582

**Table 3:** The relative weight of model 2 (plain + enhanced) CT characteristics for predicting malignancy.

Feature	Coef	Mean	SD	Relative_to_max
Heterogeneous_enhancement	1.8129	0.6709	0.4699	1
Vascular_invasion	0.9249	0.1429	0.3499	0.5043
Pleural_indentation	0.5614	0.5867	0.4924	0.2839
Age	0.5085	55.7806	9.8904	0.2792
Lobulated	0.4041	0.4031	0.4905	0.2275
Air_Bronchograms	0.3918	0.1148	0.3188	0.2114
Location_2 (0 = Peripheral;1 = Central)	0.3338	0.0969	0.2959	0.1512
Gender (0 = woman; 1 = man)	0.3022	0.5332	0.4989	0.1637
Diameter(mm)	0.2285	15.7395	4.9086	0.117
Left_upper	0.2274	0.2449	0.43	0.1161
Postobstructive_Pneumonia	0.1317	0.148	0.3551	0.0792
Cavitation	0.0963	0.1403	0.3473	0.0645
Left_lower	0.0602	0.1837	0.3872	0.0195
Plain_CT_Value	0.0056	21.602	16.8444	0.0031
Right_lower	− 0.0768	0.1888	0.3913	− 0.0599
Right_upper	− 0.1223	0.3112	0.463	− 0.0645
Enhanced_CT_Value	− 0.1492	53.0365	30.8263	0.0315
Enhancement_rate	− 0.2405	2.2591	7.5389	− 0.1541
Uniform_enhancement	− 0.2553	0.051	0.22	− 0.1354
Necrosis	− 0.2832	0.0638	0.2444	− 0.1495
Calcification	− 0.3336	0.1173	0.3218	− 0.1872
Smooth	− 0.4178	0.2321	0.4222	− 0.2141
Satellite_Nodules	− 0.4785	0.0816	0.2738	− 0.2581
Shape (0 = round/oval; 1 = irregular)	− 0.7041	0.3597	0.4799	− 0.3814
Fat	− 0.9425	0.0791	0.2699	− 0.472

### Classification performance of the two models

**Fig. 1:**
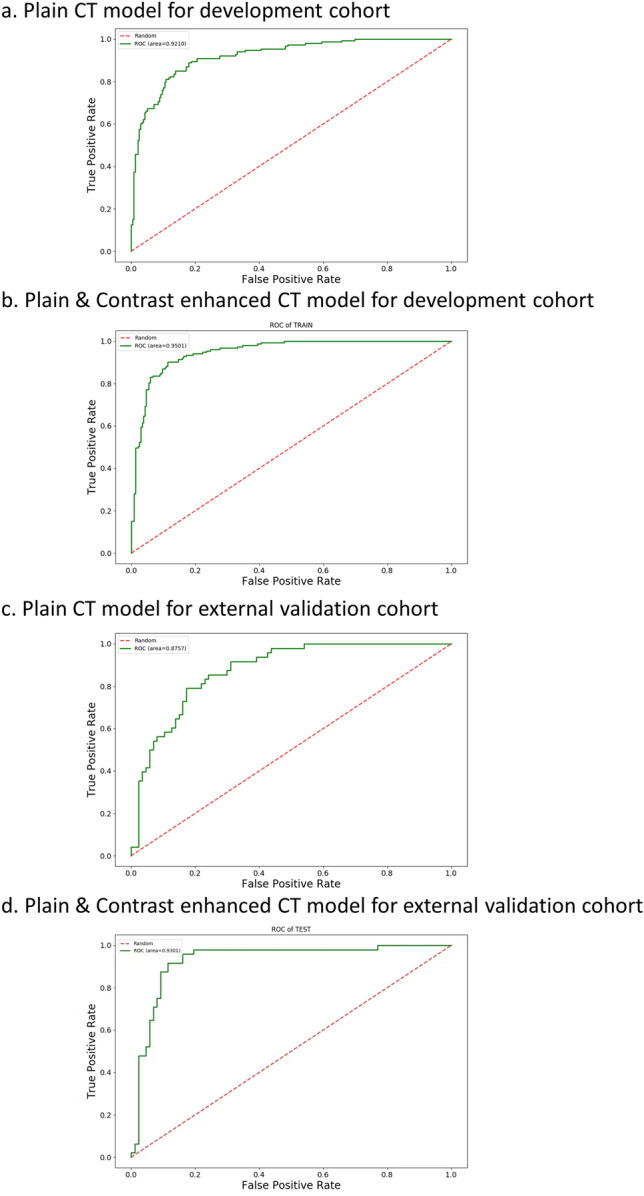
The AUC curves of model 1 and model 2 in the two cohorts.

**Fig. 2:**
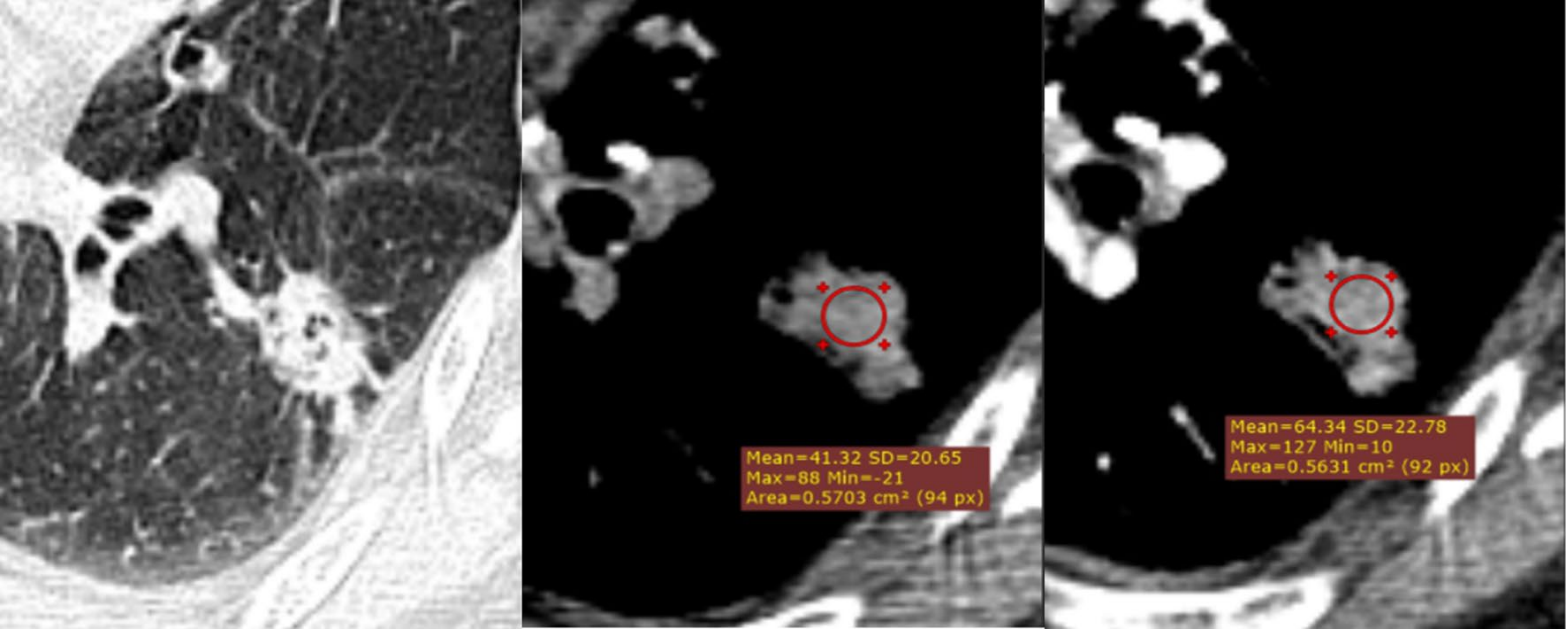
Examples of malignant case A 58-year-old female patient with a 25mm diameter, peripheral, irregular, lobulated, cavitation, pleural indentation lesion located in the left lower lobe. The mean density of the nodule was 41 HU on plain CT and 64 HU after contrast (heterogeneous enhancement). Model malignancy classification scores: Malignant (73.52%); Benign (26.48%). Histology: Adenocarcinoma.

**Fig. 3:**
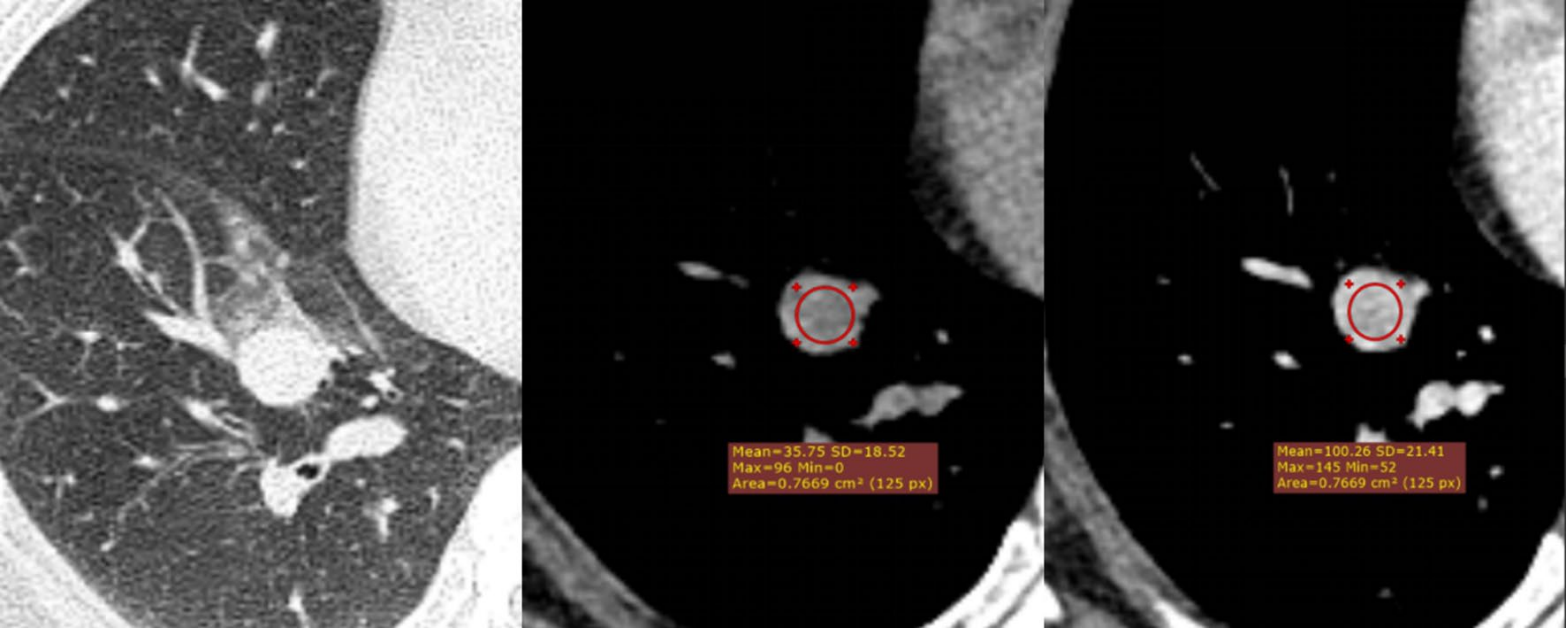
Examples of benign case A 50-year-old female patient with a 16 mm diameter, peripheral, round, smooth lesion located in the right lower lobe. The mean density of the nodule was 36 HU on plain CT and 100 HU after contrast (uniform enhancement). Model malignancy classification scores: Malignant (0.08%); Benign (99.92%). Histology: Sclerosing pneumocytoma.

The present study reports on the performance of two CT-based models in a training cohort. The model 1 (plain CT only) demonstrated a mean sensitivity, specificity, accuracy, PPV, NPV, and AUC (95%CI) of 0.85, 0.84, 0.84, 0.77, 0.90, and 0.92 (95%CI, 0.89–0.95), respectively. Model 2 (plain + contrast CT) exhibited a mean sensitivity, specificity, accuracy, PPV, NPV, and AUC (95%CI) of 0.91, 0.87, 0.88, 0.81, 0.94, and 0.95 (95%CI, 0.93–0.97), respectively. In the external validation cohort, the mean sensitivity, specificity, accuracy, PPV, NPV, and AUC (95%CI) of model 1 (plain CT only) were 0.79, 0.78, 0.79, 0.67, 0.87, and 0.88 (95%CI, 0.82–0.93). Model 2 (plain + contrast CT) were 0.88, 0.91, 0.90, 0.84, 0.93, 0.93 (95%CI, 0.88–0.98), respectively. The detailed prediction performance of model 1 and model 2 in two cohorts is shown in Table 4. The AUC curves for the two models in the two cohorts are shown in Fig. 1. Model 2 (plain + contrast CT) showed the highest diagnosis performance, we modeled the model as logistic distribution in the equation shown in Supplementary Materials 1. Examples of the cases are shown in Figs. 2 and 3.

**Table 4:** Classification performance of plain CT-based model and plain & enhanced CT-based model for nodule malignancy in the two cohorts.

Cohort		Prediction	Pathological results (No. %)				Predictive value(NPV/PPV)	AUC (95% CI)	Accuracy (%)
			Benign		Malignant				
Training cohort	Unenhanced CT model	Benign	201(84.1%)	T.N	23(15.0%)	F.N	89.7%	0.92 (0.89–0.95)	84.4
		Malignant	38(15.9%)	F.P	130(85.0%)	T.P	77.4%		
	Contrast-enhanced CT model	Benign	207(86.6%)	T.N	14(9.2%)	F.N	93.7%	0.95 (0.93–0.97)	88.3
		Malignant	32(13.4%)	F.P	139(90.5%)	T.P	81.3%		
External Validation Cohort	Unenhanced CT	Benign	68(78.2%)	T.N	10(20.8%)	F.N	87.2%	0.88 (0.82–0.93)	78.5
		Malignant	19(21.8%)	F.P	38(79.2%)	T.P	66.7%		
	Contrast-enhanced CT	Benign	79(90.8%)	T.N	6(12.5%)	F.N	92.9%	0.93 (0.88–0.98)	89.6
		Malignant	8(9.2%)	F.P	42(87.5%)	T.P	84.0%		

T.P, true positive; T.N, true negative; F.P, false-positive; F.N, false-negative; NPV, negative predictive value; PPV, positive predictive value; AUC, Area Under Curve.

## Discussion

Our dual-center study demonstrated that model 2 (plain + contrast CT ) with twenty-five CT variables which include four contrast-enhanced variables (enhanced CT value, enhancement rate, uniform enhancement, heterogeneous enhancement) had better prediction performance (0.93 [95%CI, 0.88–0.98]) than model 1 (plain CT only) (0.88 [95%CI, 0.82–0.93]) for solitary solid pulmonary nodules.

Various models, including the Mayo Clinic model, the Veterans Affairs (VA) model, and the Brock model (PanCan model), have been developed utilizing clinical and CT characteristics to assess the malignancy of lung nodules^20–22^. The Mayo Clinic model identified age, smoking history, cancer history, nodule diameter, spiculation, and upper lobe as predictors of malignant nodules^20^. The Brock model was developed to detect malignancy in nodules through low-dose CT screenings, utilizing predictors such as age, sex, family history of lung cancer, nodule location, emphysema, nodule size, and spiculation^21^. The Veterans Affairs utilized logistic regression to design a model specifically for solitary nodules, estimating the likelihood of malignancy based on factors such as age, nodule diameter, smoking history, and time since quitting smoking^22^. However, prior research has demonstrated that while these models exhibit strong performance on their respective datasets, their utility for detecting large lung nodules is limited, necessitating optimization of model characteristics before clinical application^23–27^. Our study showed similar relative variables for predicting nodule malignancy in the model, like nodule location, nodule diameter, shape, age, and gender. Furthermore, our model incorporates a greater number of semantic features, such as air bronchogram, pleural indentation, vascular invasion, postobstructive pneumonia, cavitation, necrosis, calcification, satellite nodules, and fat, as well as enhancement characteristics such as enhanced CT value, enhancement rate, uniform enhancement, and heterogeneous enhancement. The importance of semantic features has already been proved by previous study^28^. Xiang et al. showed six radiological characteristics (diameter, lobulation, calcification, spiculation, pleural indentation, and vascular invasion) were adopted as important predictors in their SVM model for the diagnosis of solid solitary pulmonary nodules with AUC 0.89. Our model 2 (plain + contrast CT) showed a higher AUC of 0.93 since we included more semantic features and enhancement characteristics (enhanced CT value, enhancement rate, uniform enhancement, heterogeneous enhancement).

The significance of CT enhancement level in the determination of malignancy in lung solid nodules has been established^18,29^. A lack of significant enhancement on contrast-enhanced CT (< 15HU) is indicative of a benign nodule. Consequently, contrast-enhanced CT has been widely utilized as the primary imaging examination technique before surgery, particularly in less developed nations^12^. Our study showed a logistic regression model based on plain CT only for predicting malignancy of solitary solid pulmonary nodules with sensitivity 0.85, specificity 0.84, and diagnostic accuracy 0.84 in the training cohort and 0.79, 0.78, 0.79 in the external validation cohort. When we added contrast-enhanced CT features into the model, it improved the diagnosis performance with a sensitivity of 0.91, specificity of 0.87, and diagnostic accuracy of 0.88 in the training cohort and 0.88, 0.91, and 0.90 in the external validation cohort. A study showed the diagnostic sensitivity, specificity, and accuracy of radiologists are approximately 0.76, 0.73, and 0.88 in a Chinese dedicated cancer hospital^24^. This means that compared with the subjective experience of radiologists, our model 2 has a 12% higher sensitivity and 18% higher specificity for diagnosing lung cancer. It effectively reduces the missed diagnosis of lung cancer and avoids excessive surgery caused by misdiagnosis. This again suggests enhanced CT could be the basis for solitary solid pulmonary nodules preoperative diagnosis especially when preoperative biopsy and PET-CT are not applicable. At the same time, due to the use of iodinated contrast agents, increases the risk of patient allergies and contrast-induced nephropathy. Developing more safe and lung cancer-specific contrast agents in the future is a direction for improving enhanced CT application.

The present study has identified certain limitations. Firstly, model 2 (plain + contrast CT) utilized in the study comprises only fundamental clinical information such as age and gender, while other factors such as smoking history, cancer history, and family history of cancer are worth considering for inclusion. Secondly, the enhanced CT values ​​used in the model are affected by scanning parameters, different contrast agent concentrations, and scanning time. Therefore, it is necessary to study the differences in enhancement characteristics between different CT equipment in the future. The study data solely comprised clinical patients, and thus, the efficacy of the model for lung cancer screening patients requires further verification. Additionally, the evaluation of the model was restricted to two datasets, and therefore, additional validation at various centers is necessary before its clinical application.

To conclude, a logistic regression model was constructed utilizing plain + contrast-enhanced CT characteristics, exhibiting superior efficacy in the assessment of malignancy in solitary solid lung nodules when compared to only plain CT-based models. The utilization of this model 2 (plain + contrast CT) enables radiologists to provide recommendations concerning follow-up or surgical intervention for preoperative patients presenting with solid lung nodules.

## Supplementary Information

Supplementary Information.

## Data Availability

The data that support the findings of this study are available upon request from the corresponding author, sxlscjy@163.com, upon reasonable request.
